# Chronic Expanding Hematoma Presenting 15 Years After Metal-on-Metal Total Hip Arthroplasty With Multiple Recurrences: A Case Report

**DOI:** 10.7759/cureus.104478

**Published:** 2026-03-01

**Authors:** José Pablo Bibiloni Lugo, Leslian Velez-Ramos, Hiram E Luigi Martinez, Jose E Martinez, Juan Bibiloni

**Affiliations:** 1 Department of Orthopaedic Surgery, Ponce Health Sciences University, Ponce, PRI

**Keywords:** arthroplasty complications, chronic expanding hematoma, metal-on-metal arthroplasty, periprosthetic mass, total hip arthroplasty

## Abstract

Chronic expanding hematoma (CEH) is an uncommon clinicopathologic entity characterized by a slowly enlarging, encapsulated hematoma that can mimic neoplasms or infection. CEH is typically associated with prior trauma or surgery and may present months to years after the inciting event. Computed tomography and magnetic resonance imaging can help define the lesion, but findings are often nonspecific; histopathologic examination is required for definitive diagnosis. Complete surgical excision, including the pseudocapsule, is generally recommended to minimize recurrence. We report a 67-year-old Hispanic male who developed progressive left hip pain and a large periprosthetic soft-tissue mass 15 years after metal-on-metal total hip arthroplasty. Repeated aspirations and cultures were negative; serum metal ion levels remained within a low range; and histopathology demonstrated an organizing hematoma without adverse local tissue reactions or malignancy. The lesion recurred multiple times, ultimately requiring repeated surgical interventions. This case highlights CEH as a rare cause of late-onset periprosthetic mass and pain after hip arthroplasty and underscores the importance of considering CEH in the differential diagnosis of late-onset periprosthetic masses when infection, adverse local tissue reactions, and malignancy have been excluded.

## Introduction

Chronic expanding hematoma (CEH) is a rare clinicopathologic entity characterized by the persistent expansion of a hematoma over months to years following an initial bleeding episode [1,2]. It is most commonly associated with trauma or prior surgical intervention and typically involves the soft tissues of the trunk or extremities [2]. First described by Reid et al., CEH is thought to result from repeated microhemorrhages within a fibrous capsule formed by chronic inflammation secondary to the degradation of blood products [1]. Although benign, CEH frequently mimics adverse local tissue reactions, infectious processes, or malignancy on imaging and clinical examination, posing a significant diagnostic challenge [3,4].

In patients with metal-on-metal total hip arthroplasty (THA), delayed periprosthetic soft-tissue masses have been described in association with adverse local tissue reactions (ALTR), including metallosis and pseudotumor formation [5,6]. These entities may present years after implantation and can demonstrate imaging and clinical features that overlap with other inflammatory or hemorrhagic processes. Accordingly, a broad differential diagnosis is often considered when evaluating periprosthetic masses in this clinical setting.

CEH has been reported in multiple anatomical locations, including the thorax and extremities; however, its occurrence in association with THA is rare [7-9]. While postoperative hematoma formation following THA is well described, reports of CEH developing decades after the index procedure remain exceedingly uncommon. Only a small number of cases describing CEH arising two decades or more after THA have been reported in the literature, predominantly involving patients of Asian descent [2,3,9-11].

We report a case of a 67-year-old Hispanic male with a history of metal-on-metal THA who developed a recurrent periprosthetic soft-tissue mass 15 years after the index procedure. Beyond its delayed presentation, this case highlights a diagnostic challenge in contemporary arthroplasty practice: differentiating a chronic hemorrhagic process from ALTR and infection in the setting of metal-on-metal hip arthroplasty. By detailing the multidisciplinary evaluation and concordant pathologic findings across multiple interventions, this report illustrates a reproducible diagnostic pitfall and underscores the need for cautious interpretation of periprosthetic masses in this high-risk population. Given the potential for progressive local tissue involvement and recurrent presentation, accurate early differentiation of these entities remains clinically relevant in patients with metal-on-metal hip arthroplasty.

## Case presentation

A 67-year-old Hispanic male with a past medical history of hypertension, hyperlipidemia, hypothyroidism, and a metal-on-metal left THA presented with a progressive pressure-like sensation in the left hip beginning approximately 15 years after the index procedure. Despite ongoing discomfort, he remained independent in activities of daily living and ambulated without assistance at the time of initial presentation.

Initial laboratory evaluation demonstrated normal plasma chromium levels and mildly elevated cobalt levels (1.2 µg/L). C-reactive protein (CRP) was mildly elevated, while the erythrocyte sedimentation rate (ESR) remained within normal limits. Complete blood count and coagulation studies were unremarkable. Laboratory findings are summarized in Table 1.

**Table 1:** Laboratory findings at initial presentation CRP: C-reactive protein; ESR: Erythrocyte sedimentation rate; PT: Prothrombin time; INR: International normalized ratio; aPTT: Activated partial thromboplastin time.

Laboratory Test	Patient Value	Reference Range	Interpretation
Cobalt	1.2 µg/L	<1.0 µg/L	Mildly elevated
Chromium	0.7 µg/L	<1.0 µg/L	Normal
C-reactive protein (CRP)	1.8 mg/dL	<0.5 mg/dL	Mild inflammatory response
Erythrocyte sedimentation rate (ESR)	8 mm/h	<20 mm/h	No significant inflammation
White blood cell count	5.58 × 10³/µL	4.23–9.07 × 10³/µL	No leukocytosis
Hemoglobin	13.40 g/dL	13.7–17.5 g/dL	Borderline low, clinically insignificant
Prothrombin time (PT)	10.70 s	10.2–13.3 s	Normal coagulation
International normalized ratio (INR)	0.956	0.870–1.130	Normal
Activated partial thromboplastin time (aPTT)	22.50 s	25.1–33.2 s	Slightly shortened, no clinical significance

**Figure 1:**
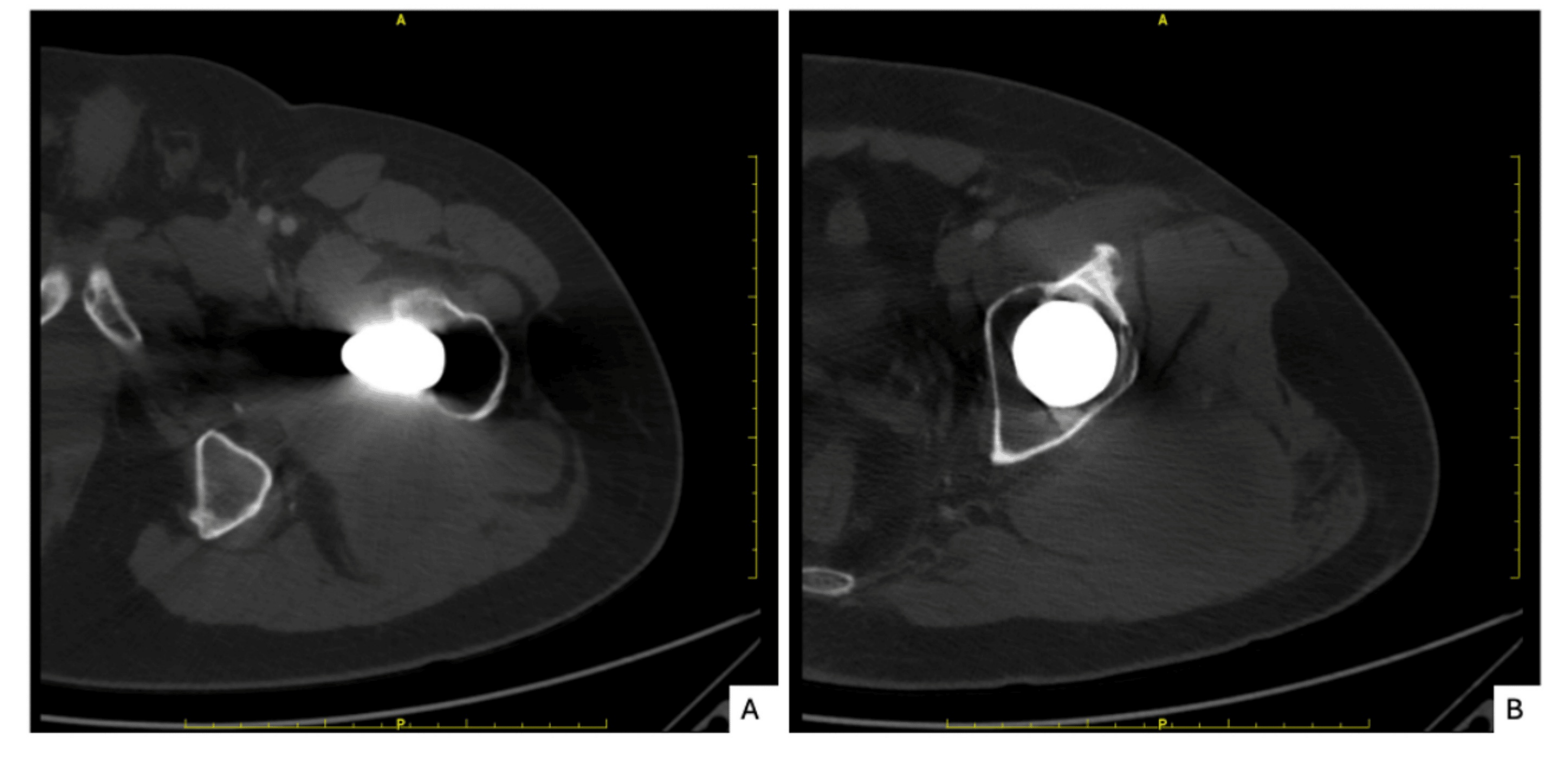
Axial contrast-enhanced CT images of the left hip (A) Axial CT image demonstrating periprosthetic lucency surrounding the femoral stem. (B) Axial CT image demonstrating posterior cortical hypertrophy and surrounding sclerosis involving the proximal femur in the trochanteric region.

Initial imaging demonstrated periprosthetic lucency involving the proximal femur without evidence of acute fracture or component loosening. Contrast-enhanced computed tomography (CT) further characterized this finding, revealing periprosthetic lucency in the trochanteric region with surrounding sclerosis and posterior cortical hypertrophy (Figure 1). A periprosthetic soft-tissue abnormality was suspected in the setting of these CT findings and the clinical presentation. Differential considerations included pseudotumor, complex periprosthetic fluid collection, chronic abscess, or neoplastic process. Subsequent nuclear imaging demonstrated no evidence of active infection.

**Figure 2:**
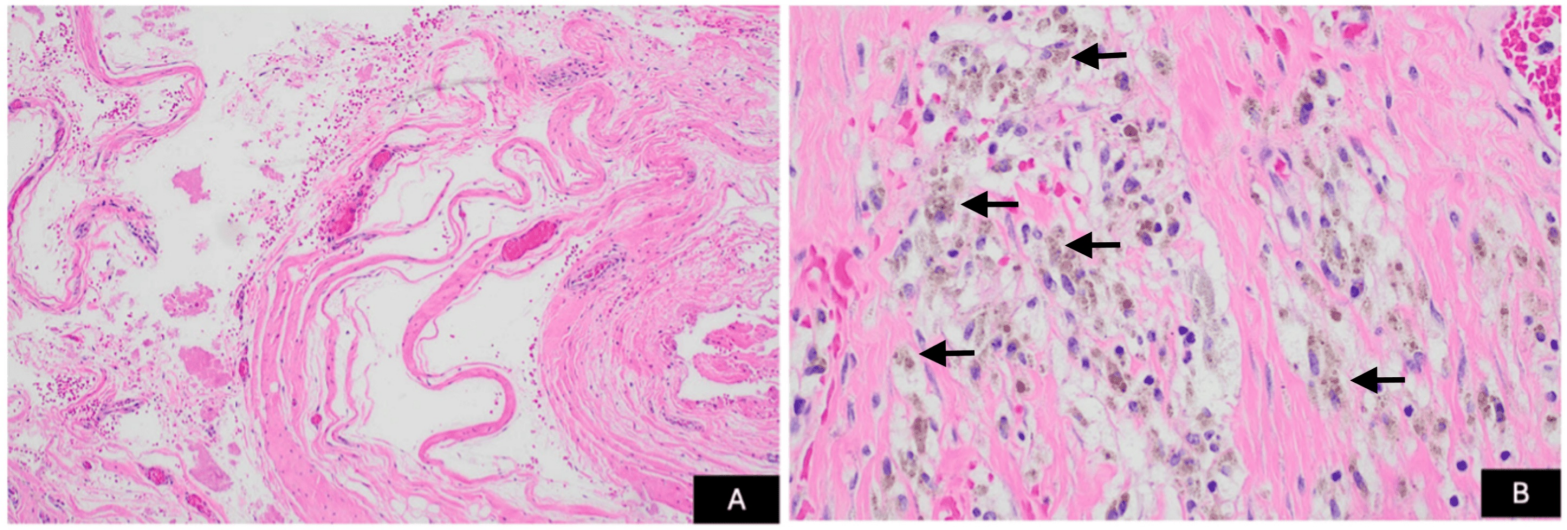
Histopathologic examination of the excised mass (A) Low-power (20x) hematoxylin and eosin (H&E) stain demonstrating dense, whorled fibrous tissue forming a pseudocapsule surrounding areas of organizing hematoma. (B) High-power (40x) H&E stain showing abundant hemosiderin-laden macrophages and chronic inflammatory infiltrate within a fibrous stroma, without epithelial lining, cytologic atypia, or malignancy, consistent with chronic expanding hematoma.

Image-guided aspiration demonstrated no bacterial growth. Given persistent symptoms and an inconclusive noninvasive evaluation, the patient underwent exploratory surgery with excision of a large grayish mass. Intraoperatively, the prosthetic components were stable, and no active bleeding source was identified. There was no gross evidence of corrosion, metallosis, component loosening, or infection, and revision arthroplasty was therefore not performed. Histopathologic evaluation revealed a chronic organizing hematoma composed of dense fibrous tissue forming a pseudocapsule, with abundant hemosiderin-laden macrophages and chronic inflammatory infiltrate, without evidence of malignancy or acute infection (Figure 2). Postoperatively, the patient experienced symptomatic improvement.

Several weeks later, the patient developed recurrent swelling associated with a draining sinus tract, requiring repeat arthrotomy with curettage and debridement. Symptoms improved following the second procedure.

**Figure 3:**
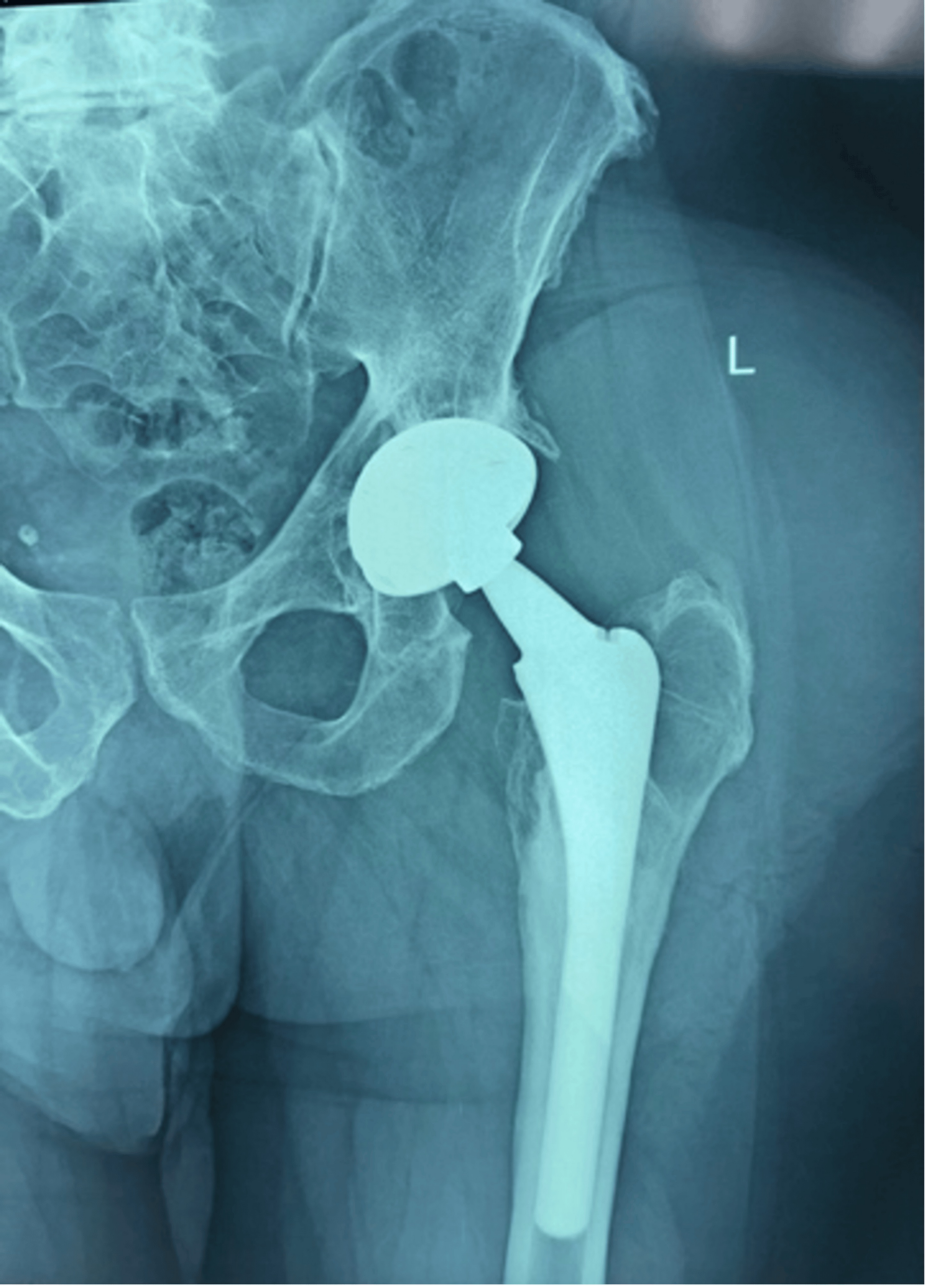
Follow-up anteroposterior (AP) radiograph of the left hip demonstrating a total hip arthroplasty with preserved component alignment and osteolytic changes involving the proximal femur and acetabulum

Approximately one year later, the patient noted a gradual recurrence of a left gluteal mass, which remained stable in size for an extended period. At subsequent follow-up, persistent drainage prompted reevaluation. Repeat radiographs demonstrated progressive osteolysis involving the proximal femur and acetabulum (Figure 3).

**Figure 4:**
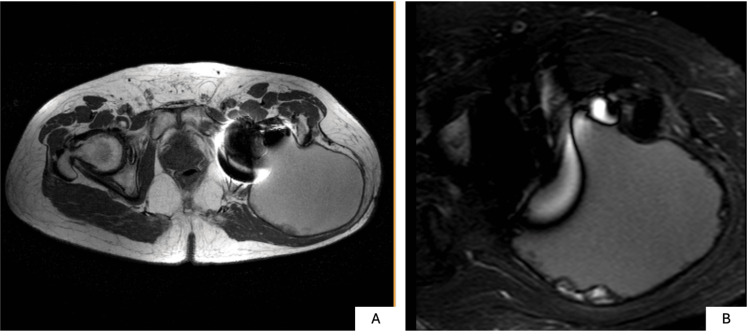
Axial MRI of the left hip obtained using metal artifact reduction sequences (MARS) (A) T1-weighted MARS image shows a well-circumscribed lesion with predominantly low to intermediate signal intensity. (B) Short tau inversion recovery (STIR) MARS image demonstrates marked hyperintensity consistent with a fluid-containing lesion, supporting the diagnosis of chronic expanding hematoma.

Magnetic resonance imaging (MRI) revealed a large multiloculated fluid collection extending through inter- and intramuscular planes of the posterolateral thigh and gluteal region, measuring 18.1 × 12.5 × 11.0 cm (Figure 4). The lesion demonstrated heterogeneous signal on T1-weighted imaging and marked hyperintensity on fluid-sensitive sequences, without neurovascular involvement.

Repeat aspiration demonstrated no bacterial or fungal growth, and cytology revealed degenerated erythrocytes and cellular debris without malignant features consistent with a resolving hematoma. Based on the chronic clinical course and imaging characteristics, a diagnosis of CEH was favored. Drainage of more than 300 cc of fluid provided temporary symptomatic relief.

Despite conservative measures, the lesion recurred, necessitating a third surgical excision. Intraoperatively, a well-encapsulated cystic mass containing dark brown fluid was identified and completely excised. Histopathologic evaluation demonstrated a pseudocyst composed of dense fibrous tissue without epithelial lining, accompanied by lymphohistiocytic infiltrate and fibrinohemorrhagic material. The postoperative course was complicated by seroma formation, requiring additional surgical management. The patient remains under close clinical surveillance for recurrence.

## Discussion

CEH is an uncommon clinicopathologic entity characterized by the gradual enlargement of a hematoma over months to decades following an initial hemorrhagic event [1]. Its pathogenesis is thought to involve a chronic inflammatory response to the breakdown of blood products, resulting in fibrous capsule formation and repeated microhemorrhage [12]. Although CEH is most commonly associated with trauma or prior surgical intervention, the precise mechanisms underlying its progressive expansion remain incompletely understood [13]. Systemic factors, including coagulopathies, have been implicated in some reported cases [10]. In contrast, our patient demonstrated normal coagulation parameters and hemoglobin levels throughout his clinical course, with no evidence of an underlying bleeding disorder.

CEH has most frequently been described in the thorax, retroperitoneum, and extremities, whereas periprosthetic involvement remains rare [7,8,14]. In the present case, CEH developed 15 years after primary metal-on-metal THA in the absence of preceding trauma or additional surgical intervention. Notably, serum cobalt and chromium concentrations remained within normal limits on repeated testing; however, normal metal ion levels do not exclude metallosis or adverse local tissue reaction, as previously reported [15]. These findings emphasize the importance of maintaining a broad differential diagnosis when evaluating delayed periprosthetic soft-tissue masses, even in patients with metal-on-metal implants.

Review of previously reported cases of CEH following THA reveals several consistent clinical patterns. Delayed presentations have been documented two to four decades after prosthesis implantation, with most patients reporting progressive pain and swelling [2,3,9-11]. Imaging frequently demonstrates large, heterogeneous masses that raise concern for pseudotumor, malignancy, or chronic abscess formation [10]. Pseudotumor formation after THA is most commonly associated with adverse reactions to metal debris, and in patients with metal-on-metal implants, evaluation with MRI and serum metal ion levels is recommended [5]. However, MRI characteristics of CEH may substantially overlap with those of pseudotumors, limiting the ability to reliably distinguish these entities on imaging alone. CEH typically demonstrates heterogeneous signal intensity on both T1- and T2-weighted sequences with a peripheral low-signal fibrous rim, whereas pseudotumors more often appear as mixed solid-cystic or predominantly solid lesions [6,16].

Consistent with prior reports, definitive diagnosis in this case was achieved only after surgical excision and histopathologic analysis. Characteristic findings include an organizing hematoma composed of dense fibrous tissue without epithelial lining, hemosiderin-laden macrophages, and chronic inflammatory infiltrates in the absence of malignant features [17,18]. In this patient, the reproducibility of histopathologic findings across multiple surgical excisions substantially strengthens the diagnostic certainty of CEH, despite the absence of a clearly identifiable inciting hemorrhagic event. Complete surgical excision of the hematoma and its surrounding capsule is generally considered the treatment of choice for CEH [10]. Given the absence of implant loosening, normal metal ion levels, and lack of histopathologic features of adverse local tissue reaction, isolated excision rather than revision arthroplasty was considered appropriate.

Nevertheless, recurrence has been described, particularly in cases of incomplete excision or persistent inflammatory stimulus, underscoring the importance of meticulous resection and close postoperative surveillance [19]. Although the precise mechanism underlying the recurrent collections observed in this case remains speculative, persistent local inflammatory stimulation within a fibrous capsule has been proposed as a contributing factor in similar cases.

Given its ability to mimic ALTR, infection, or malignancy both clinically and radiographically, CEH should remain an important consideration in the evaluation of delayed periprosthetic masses, particularly when imaging reveals an encapsulated lesion containing blood products, and laboratory and microbiological studies are unremarkable. Histopathologic examination remains essential for definitive diagnosis and for distinguishing CEH from pseudotumor or neoplastic processes. Early recognition of this rare entity may prevent unnecessary oncologic interventions and guide appropriate surgical management. The present case is notable for multiple recurrences requiring repeated surgical interventions, features that are rarely described in the existing literature. Additionally, this appears to represent one of the few reported cases of CEH developing many years after THA in a Hispanic patient, contributing to the limited demographic data available for this uncommon complication.

## Conclusions

This case represents a rare and delayed presentation of a CEH occurring 15 years after THA. Given its ability to mimic more concerning entities such as pseudotumors, CEH should be included in the differential diagnosis of late-onset periprosthetic soft-tissue masses. Definitive distinction between CEH and pseudotumor relies on histopathologic examination of the excised lesion. Early recognition and complete surgical excision are important to reduce the risk of recurrence and optimize outcomes. The present case is notable for multiple recurrences within a relatively short period, underscoring the importance of close clinical and radiographic follow-up in affected patients. Further studies are needed to better elucidate the pathogenesis of CEH and to define optimal management strategies in the setting of orthopedic implants.
